# Effect of norepinephrine infusion on hepatic blood flow and its interaction with somatostatin: an observational cohort study

**DOI:** 10.1186/s12871-022-01741-2

**Published:** 2022-07-02

**Authors:** Jurgen van Limmen, Xavier Iturriagagoitia, Marilie Verougstraete, Piet Wyffels, Frederik Berrevoet, Luís Filipe Abreu de Carvalho, Stefan De Hert, Luc De Baerdemaeker

**Affiliations:** 1 Department of Anesthesiology and Perioperative Medicine, Ghent University Hospital, Corneel Heymanslaan 10, 9000 Ghent, Belgium; 2 Department of General and Hepatic-Pancreatico-Biliary Surgery and Liver Transplantation, Ghent University Hospital, Corneel Heymanslaan 10, 9000 Ghent, Belgium

**Keywords:** Norepinephrine, Liver circulation, Hepatic blood flow, Somatostatin

## Abstract

**Background:**

Norepinephrine (NE) is a α_1_-adrenergic mediated vasopressor and a key player in the treatment of perioperative hypotension. Apart from modulating systemic hemodynamics, NE may also affect regional blood flow, such as the hepatic circulation, which contains a wide variety of adrenergic receptors. It may alter regional vascular tonus and hepatic blood flow (HBF) by reducing portal vein flow (PVF) or hepatic arterial flow (HAF). The aim of this study was to assess the effects of NE on HBF.

**Methods:**

Patients scheduled for pancreaticoduodenectomy were included. All patients received standardized anesthetic care using propofol and remifentanil and were hemodynamically stabilized using a goal-directed hemodynamic strategy guided by Pulsioflex™. On surgical indication, somatostatin (SOMATO) was given to reduce pancreatic secretion. HBF measurements were performed using transit-time ultrasound (Medistim™). Baseline hemodynamic and HBF measurements were made after pancreatectomy, at T1. Afterwards, NE infusion was initiated to increase mean arterial pressure (MAP) by 10 – 20% of baseline MAP (T2) and by 20 – 30% of baseline MAP (T3). HBF and hemodynamic measurements were performed simultaneously at these three time-points.

**Results:**

A total of 28 patients were analyzed. Administration of NE significantly increased MAP but had no effect on cardiac index. NE infusion reduced total HBF in all patients (*p* < 0.01) by a reduction HAF (*p* < 0.01), while the effect on PVF remained unclear. Post-hoc analysis showed that SOMATO-treated patients had a significant lower PVF at baseline (*p* < 0.05), which did not change during NE infusion. In these patients, reduction of total HBF was primarily related to a reduction of HAF (*p* < 0.01). In untreated patients, NE infusion reduced total HBF both by a reduction HAF (*p* < 0.01) and PVF (*p* < 0.05).

**Conclusion:**

Administration of NE reduced total HBF, by decreasing HAF, while the effect on PVF remained unclear. SOMATO-treated patients had a lower PVF at baseline, which remained unaffected during NE infusion. In these patients the decrease in total HBF with NE was entirely related to the decrease in HAF. In SOMATO-untreated patients PVF also significantly decreased with NE.

**Trial registration:**

Study protocol EC: 2019/0395.

EudraCT n°: 2018–004,139-66 (25 – 03 – 2019).

Clin.trail.gov: NCT03965117 (28 – 05 – 2019).

## Introduction

The association between maintaining adequate perioperative blood pressure and postoperative preservation of organ function is well documented [1, 2]. Use of vasoconstrictive medication is one of the key elements in treating perioperative hypotension. However, their administration may affect regional perfusion.

Norepinephrine (NE) is a commonly used vasopressor during goal-directed hemodynamic therapy (GDHT) to increase blood pressure [3]. However, it may also affect systemic hemodynamic effects by its inotropic effect. In addition, NE induces venoconstriction and arterial vasoconstriction, by which it increases respectively preload and afterload [4–6].

The splanchnic system is an important blood reservoir and redistribution of blood from the splanchnic circulation by venoconstriction, highly contributes to venous return, cardiac output and systemic hemodynamics [7]. In addition to these systemic hemodynamic effects, NE also exerts regional hemodynamic effects that may affect splanchnic blood flow.

Hepatic blood flow contributes to the splanchnic circulation. Indeed, regulation of splanchnic blood flow is determined by splanchnic vascular resistance sites, which are located mainly in the pre-portal arterial vasculature, by hepatic arterial vasculature, and by portal venous vasculature [8–11]. These resistances are regulated by extrinsic control mechanism such as the sympathetic nervous system and circulating humoral factors acting on a wide variety of adrenergic receptors [12, 13]. NE interacts with these adrenergic receptors which may result in changes in cardiac output, systemic and splanchnic vascular tone and thus eventually splanchnic blood flow [14].

The aim of the study was to assess the effect of NE infusion on HBF and systemic hemodynamic variables in hemodynamically stabilized patients.

## Methods

### Design and patients

The study was approved by the ethics committee of the Ghent University Hospital (EC: 2019/0395) and registered under EudraCT number: 2018–004,139-66 (25 – 03 – 2019) and at Clin.trail.gov number NCT03965117 (28 – 05 – 2019). After written informed consent, patients of both genders aged between 18 and 80 years, scheduled for pancreaticoduodenectomy (Whipple`s procedure) and with an American Society of Anesthesiologist (ASA) physical status I to III were included. Exclusion criteria were allergy to norepinephrine, renal insufficiency (serum creatinine > 2 mg dL^−1^), severe heart failure (ejection fraction < 25%), hemodynamic instability, atrial fibrillation, sepsis, body mass index > 40 kg m^−2^, severe coagulopathy (INR > 2), thrombocytopenia (< 80 × 10^3^ μL^−1^), end stage liver disease or pregnancy and breastfeeding women.

The methodology of the measurements has already been described in a previous publication [15]. After pancreatectomy, the HBF measurements were performed. Blood flow measurements were obtained at the hepatic artery (HAF) and portal vein (PVF) using perivascular ultrasound transit time flow probes (TTFM, Medi-Stim AS, Oslo, Norway) [16]. Total HBF was calculated using PVF and HAF measurements. These flow measurements were related to the hemodynamic variables, which were measured simultaneously. During the observation period, 3 flow measurements were performed by the surgeon, at predefined time-points in which MAP was gradually increased by NE infusion. Systemic hemodynamic variables as well as HBFs and pressures were recorded at 3 time-points. A baseline measurement was performed after pancreatectomy (T1). NE infusion was started after T1 to increase baseline MAP by 10 – 20%. When target MAP was reached, a second measurement was performed (T2). After this measurement, NE was further increased to raise MAP by 20 – 30% of baseline MAP. When the target MAP was reached, a final set of measurements were performed before surgical reconstruction was continued (T3).

The primary objective was to assess – in hemodynamically stabilized patients – the effect of NE on hepatic blood flow (HBF) and hepatic vascular pressures such as caval pressure (P_Cava_) and portal pressure (P_Porta_). The secondary objective of the study was to evaluate the effects of NE infusion on systemic hemodynamic variables such as systemic vascular resistance indexed (SVRi), portal venous resistance indexed (PVRi), cardiac index (CI), blood loss and total fluid administration. Post-hoc analysis was performed on patients who received somatostatin (SOMATO) to evaluate its effect on HBF and the interaction with NE.

### Anesthetic procedure

All patients received standard anesthesia care according to the departmental written protocol. Before induction of anesthesia, an epidural catheter was placed for postoperative analgesia but only used at the end of surgery, after all experimental measurements were performed. Induction and maintenance of general anesthesia was obtained using target-controlled infusion (TCI) of propofol (Schnider model), starting at an effect site concentration of 5.0 mcg ml^−1^. Depth of anesthesia was monitored using Bispectral Index (BIS™, Covidien, MA, USA) and propofol was titrated to remain a BIS value between 40 and 60. For intraoperative analgesia, TCI remifentanil (Minto model) was used. TCI remifentanil was started at an effect site concentration of 5 ng ml^−1^ and titrated according to heart rate and blood pressure. Neuromuscular blockade was achieved using rocuronium, 1 mg kg^−1^ ideal body weight at induction and intermittent boluses during surgery. An additional bolus of rocuronium 10 mg was given before the experimental measurements. After tracheal intubation and lung recruitment, mechanical ventilation was started with a tidal volume 8 – 10 ml kg^−1^ ideal body weight, respiratory rate 14 – 16 min^−1^ and a positive end-expiratory pressure of 5 cmH_2_O. Arterial blood gas analysis was used to adjust ventilatory settings.

Hemodynamic monitoring was performed using a PiCCO catheter (Maquet, Getinge Group, Germany) which was placed in the left femoral artery and a central venous catheter which was placed in the right jugular vein. All patients received a standardized goal-directed hemodynamic therapy (GDHT). A baseline crystalloid infusion (Plasmalyte A, Baxter S.A., Lessines, Belgium) of 3 ml kg^−1^ h^−1^ was administered. The hemodynamic goal was a CI > 2.0 L min^−1^ m^−2^ with a MAP > 60 mmHg and a pulse pressure variation (PPV) < 12%. When PPV was > 12% a bolus of 200 ml crystalloids (Plasmalyte A, Baxter S.A., Lessines Belgium) was administered. In case of bleeding, 200 ml colloid (Volulyte A, Fresenius Kabi NV, Schelle Belgium), was administered. Intraoperative hypotension was defined at MAP < 60 mmHg. When CI was > 2.0 L min^−1^ m^−2^ in the presence of a MAP < 60 mmHg, a NE infusion was started at 0.1 mcg kg^−1^ min^−1^ and titrated according to the MAP. To temporarily bridge the latency of effect the NE infusion, boluses of ephedrine 3 mg were administered when heart rate (HR) was less than 60 beats per minute or phenylephrine 100 mcg, if HR > 60 beats min^−1^. At the discretion of the surgeon, some patients received SOMATO, prior to the pancreatic resection, to reduce pancreatic secretion. In that case, SOMATO 240 mcg bolus was administered, followed by a continuous infusion of SOMATO of 6 mg per 24 h. For postoperative analgesia, all patients received clonidine 150 mcg and magnesium 2 g intravenously after opening of the abdominal cavity. At the end of surgery, all patients received 1 g paracetamol and 10 – 15 ml ropivacaine 0.15% epidurally. For prevention of postoperative nausea and vomiting, dexamethasone 5 mg was administered before induction of anesthesia and ondansetron 4 mg was given at the end of surgery. A nerve stimulator was used to assess evoked muscular response with train-of-four ratio. Reversal of neuromuscular block was achieved using sugammadex according to train-of-four ratio.

### Measurements

Hemodynamic variables were measured and recorded using Pulsioflex™ (Maquet, Getinge Group, Germany). After placement of the 4 or 5-Fr arterial catheter in the femoral artery, the pulse contour analysis was calibrated using 3 boluses of 20 ml of cold saline. The hemodynamic variables used were HR, central venous pressure (CVP), MAP, CI and PPV. Calibration was repeated before experimental measurements were initiated.

TTFM was used to measure HAF and PVF. Different probe sizes were used according to the type and size of the vessel (range 2 – 12 mm) and blood flow was expressed in ml min^−1^. At the same time, the pulsatility index (PI) was calculated. It quantifies pulsatility of a blood flow wave which represents vascular resistance of the blood vessel downstream.

After pancreatectomy, baseline MAP was recorded at T1. NE infusion (60 mcg.ml^−1^ solution) was started at 0,1 mcg kg^−1^ min^−1^ to increase MAP by 10 – 20% of baseline MAP (T2) and 20 – 30% of baseline MAP (T3). NE infusion was given through a dedicated lumina of the central line and a continuous infusion of saline at 50 ml.h^−1^ was used as drive line. NE was titrated according to MAP with 0.5 ml.h^−1^. Before each measurement, a stabilization period of 5 min was allowed, to ensure steady state hemodynamic conditions. At each specific timepoint hepatic blood flows and pressures were measured together with the systemic hemodynamic data. Simultaneously with flow measurements, pressure measurements were performed in the portal and caval vein. A 25-gauge needle was placed directly in the vein and connected to a pressure transducer. All measurements were performed during apnea to minimize the effect of ventilation.

HBF was indexed to body surface area and calculated as % of CO. Portal venous resistance (PVRi) was calculated and indexed to body surface area:

$$PVRi=\left(\frac{{P}_{Porta}-CVP}{{PVF}_{i}}\right)\times 80$$

To address the patient fluid status, mean circulatory filling pressure (Pms) and heart performance (Eh) were calculated [17]:

Pms is determined by CVP, MAP and CO and expressed in mmHg:

$$Pms=0.96\;CVP+0.04\;MAP+c CO$$

Factor c is depending on length, weight and age:

$$c=\frac{\left(0.038 \left(94.17+0.193 \times age\right)\right)}{(4.5 \times {0.99}^{\left(age-15\right)} \times 0.007184 \times {length}^{0.725}\times {weight}^{0.425})}$$

The heart performance (Eh) can be calculated according to following formula:

$$Eh= \frac{\Delta (Pms-CVP)}{\Delta Pms}$$

Vascular tone of the hepatic artery in the different experimental conditions, was quantified by calculating the vascular conductance [18]:

$$Conductance= \frac{{HAF}_{i}}{MAP}$$

### Statistical analysis

For sample size calculation, a previous pilot trial with similar methodology was used [15]. Based on this publication, a 15% reduction in HAF and PVF was considered clinically significant. G*Power 3.1.9.2 [19] was used to calculate the sample size. For an alpha error of 5% and a beta error of 20%, we necessitated 28 patients to detect a flow reduction of 15%. We used linear mixed modelling to compare HBF, HAF and PVF at the 3 study points. Patient identity was used as random effect (random intercept), to account for the repeated measures. The study points were used as fixed effect. Logarithmic transformation was used to meet the assumption of normal distribution of the residuals.

To assess a potential effect of the concomitant administration of SOMATO on the effect of NE, a post hoc analysis was performed. Patients were divided based on presence (Group S, *n* = 20) or absence (Group NS, *n* = 8) of SOMATO administration. The statistical model was updated by adding these groups as interaction factor to the fixed effect. All statistical tests were performed using R (version 3.3.3) [20]. Lme4-package (v 1.1–23) and car-package (v 3.0–9) were used for linear mixed modelling and to determine the optimal transformation within the Box-Cox family, respectively.

## Results

### Patient characteristics

**Fig. 1:**
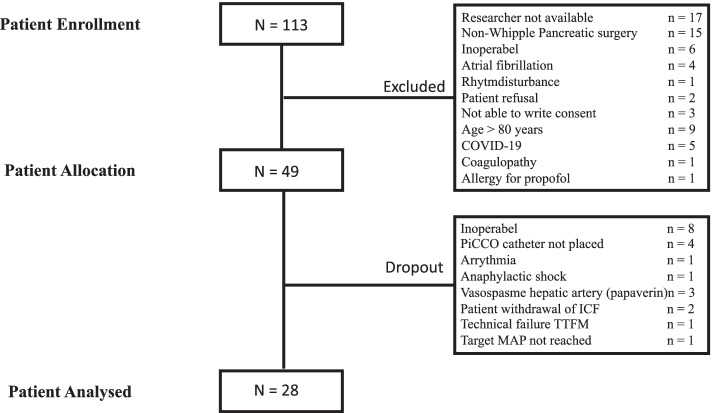
STROBE diagram

Between 28/05/2019 and 19/10/2020 a total of 113 patients were screened for eligibility. Registration of the first patient (number 01) was done on 29/05/2019. A total of 49 patients were included of which 21 dropped out, due to inoperability (*n* = 8), inability to place a PiCCO catheter due to atheromatous plaques in the femoral artery (*n* = 4), intraoperative arrythmia (*n* = 1), intraoperative anaphylactic shock with hemodynamic instability (*n* = 1), vasospasm of the hepatic artery requiring papaverine (*n* = 3), failure of the technical device (TTFM) (*n* = 1), patient withdrawal prior to inclusion (*n* = 2) and target MAP not reached (*n* = 1). Figure 1. Finally, data from a total of 28 patients were analyzed. On surgical indication, 20 of these patients were treated with SOMATO. To analyze the potential effect of SOMATO on the effects of NE, the total group of 28 patients was post hoc divided in those receiving SOMATO (group S, *n* = 20) and those who were not treated with SOMATO (group NS, *n* = 8). Patient characteristics are listed in Table 1. There were no differences in these characteristics between patients treated with and without SOMATO.

**Table 1:** Patient characteristics

	Group (*n* = 28)	Group S (*n* = 20)	Group NS (*n* = 8)
Male / Female ratio	14 / 14	9/11	5/3
Age (years)	58 (13)	56 (13)	62 (12)
Length (cm)	170 (8.2)	170 (8.6)	171 (7.5)
Weight (kg)	72.5 (13.0)	74.6 (13.2)	67 (11.5)
BMI	25.0 (3.8)	25.9 (3.6)	22.8 (3.5)
Systolic BP (mmHg)	129 (18)	131 (19)	123 (17)
Diastolic BP (mmHg)	76 (8)	72 (7)	78 (8)
MAP (mmHg)	94 (10)	96 (10)	89 (9)
HR (bpm)	75 (10)	72 (10)	80 (10)
ASA I / II / III	2 / 19 / 7	1 / 14 / 5	1 / 5 / 2
Smoker (F / N / Y)	9 / 11 / 8	7 / 7 / 6	2 / 4 / 2
Beta-blocking agent (Y / N)	2 / 26	1 / 19	1 / 7
ACE-inhibitor (Y / N)	4 / 24	2 / 18	2 / 6
Duration of surgery (min)	567 (84)	575 (75)	548 (107)

Data are expressed in mean (SD)

*BMI* Body Mass Index, *MAP* Mean Arterial blood Pressure, *HR* Heart Rate, *ASA* American Society of Anesthesiologist physical status

### Intraoperative characteristics

Intraoperative characteristics are summarized in Table 2. Anesthesia and fluid needs were similar in both groups as were also urinary output and blood loss. Only 1 patient necessitated blood transfusion of 2 units packed red blood cells.

**Table 2:** Intraoperative characteristics

	**Total Group (*n* = 28)**	**Group S** **(*n* = 20)**	**Group NS** **(*n* = 8)**
Crystalloids (ml)	4184 (1594)	4197 (1539)	4151 (1836)
Crystalloids (ml.kg^−1^.h^−1^)	6.1 (1.6)	5.9 (1.6)	6.6 (1.7)
Colloids (ml)	182 (288)	165 (313)	225 (225)
Estimated blood loss (ml)	342 (220)	339 (240)	350 (175)
Urinary Output (ml)	790 (445)	762 (381)	859 (602)
Urinary Output (ml.kg^−1^ h^−1^)	1.2 (0.7)	1.1 (0.7)	1.3 (0.6)
Ephedrine (mg)	10.5 (7.9)	9.3 (8.0)	13.5 (7.5)
Phenylephrine (mcg)	0.27 (0.31)	0.24 (0.33)	0.34 (0.28)
Propofol (mg)	3886 (997)	4052 (938)	3470 (1082)
Propofol (mg.kg^−1^.h^−1^)	5.7 (0.8)	5.7 (0.9)	5.6 (0.6)
Remifentanil (mcg)	4575 (1420)	4797 (1479)	4022 (1164)
Remifentanil (mcg.kg^−1^.min^−1^)	0.11 (0.02)	0.11 (0.02)	0.11 (0.03)
Duration of surgery (min)	567 (84)	575 (75)	548 (107)

Data are expressed in mean (SD)

### Systemic hemodynamic measurement

Data are summarized in Table 3. NE significantly increased MAP and SVRi, similarly in both groups. CI, CVP, HR, PVRi, Pms and Eh remained unchanged. NE infusion was started after T1 measurements and increased to 0.06 mcg kg^−1^ min^−1^ (SD 0.03 mcg kg^−1^ min^−1^) at T2 and 0.101 mcg kg^−1^ min^−1^ (SD 0.05 mcg kg^−1^ min^−1^) at T3. All patients met the pre-defined hemodynamic targets and none of the patients necessitated NE before the experimental measurements. Both ephedrine and phenylephrine were used to counteract post-induction hypotension (respectively 10.5 mg (SD 7.9 mg) and 270 mcg (SD 310 mcg). All patients were hemodynamically stabilized by the time of the first measurement. The pre-defined hemodynamic targets were met at baseline T1 which can be found in Table 3.

**Table 3:** Hemodynamic data

Variable	Time-Point	Total group (*n* = 28)	Somatostatin	
			**Yes (*n* = 20)**	**No (*n* = 8)**
MAP (mmHg)	T1	73 (10)	73 (11)	72 (7)
	T2	84 (10)^#^	85 (11)^#^	83 (7)^#^
	T3	93 (12)^#^	93 (13)^#^	92 (10)^#^
HR (bpm)	T1	78 (11)	77 (11)	80 (11)
	T2	75 (11)	74 (11)	78 (10)
	T3	75 (12)	75 (13)	75 (10)
CVP (mmHg)	T1	5 (3)	5 (3)	5 (4)
	T2	5 (3)	5 (3)	6 (4)
	T3	5 (3)	5 (3)	6 (4)
CI (L.min^−1^.m^−2^)	T1	3.1 (0.5)	3.1 (0.6)	3.1 (0.4)
	T2	3.1 (0.5)	3.1 (0.6)	3.1 (0.3)
	T3	3.2 (0.5)	3.2 (0.6)	3.2 (0.3)
SVRI (dyn.sec.cm^−5^.m^−2^)	T1	5698 (775)	5729 (844)	5621 (613)
	T2	6574 (820)^#^	6624 (895)^#^	6448 (628)^#^
	T3	7235 (963)^#^	7260 (1039)^#^	7172 (798)^#^
PVRi (dyn.sec.cm^−5^ m^−2^)	T1	1.4 (1.4)	1.5 (1.5)	1.2 (1.1)
	T2	1.7 (1.9)	1.8 (2.1)	1.4 (1.1)
	T3	1.4 (1.4)	1.2 (.09)	1.9 (2.2)
Pms (mmHg)	T1	12 (3)	12 (3)	12 (3)
	T2	13 (3)	13 (3)	13 (4)
	T3	13 (3)	13 (3)	14 (3)
Eh	T1	0.63 (0.20)	0.64 (0.19)	0.63 (0.23)
	T2	0.62 (0.18)	0.62 (0.16)	0.62 (0.22)
	T3	0.63 (0.16)	0.63 (0.16)	0.61 (0.17)
PPV	T1	10 (3)	10 (3)	11 (4)
	T2	9 (3)	8 (3)	10 (4)
	T3	8 (3)	8 (3)	8 (3)
pH	T1	7.32 (0.06)	7.31 (0.06)	7.34 (0.07)
	T2	7.31 (0.05)	7.31 (0.05)	7.33 (0.06)
	T3	7.31 (0.05)	7.30 (0.05)	7.32 (0.06)
Lactate (mmol.L^−1^)	T1	1.3 (0.8)	1.4 (0.9)	1.1 (0.4)
	T2	1.3 (0.8)	1.4 (0.9)	1.1 (0.4)
	T3	1.3 (0.8)	1.4 (0.9)	1.1 (0.3)
Noradrenaline (mcg kg^−1^ min^−1^	T1	0 (0.01)	0 (0.01)	0 (0.01)
	T2	0.06 (0.03)^#^	0.05 (0.03)^#^	0.06 (0.04)^#^
	T3	0.101 (0.05)^#^	0.102 (0.06)^#^	0.100 (0.05)^#^

Data are expressed in mean (SD)

*MAP* Mean Arterial Pressure, *HR* Heart Rate, *CVP* Central Venous Pressure, *CI* Cardiac Index, *SVRI* Systemic Vascular Resistance Indexed, *Pms* mean circulatory filling pressure, *Eh* heart performance, *PPV* Pulse Pressure Variation

Significant differences are marked as * for significant between group difference and ^#^ for significant within group difference, compared to T1 (*p* < 0.05)

### Hepatic blood flow measurements

Data are summarized in Table 4. NE dose-dependently reduced total HBFi from baseline 548 ml min^−1^ m^−2^ (SD 182 ml min^−1^ m^−2^) to 465 ml min^−1^ m^−2^ (SD 149 ml min^−1^ m^−2^) at T2 (*p* < 0.01) and 458 ml min^−1^ m^−2^ (144 ml min^−1^ m^−2^) at T3 (*p* < 0.01). This reduction is the result of a significant reduction in HAFi from 215 ml min^−1^ m^−2^ (SD 118 ml min^−1^ m^−2^) at T1 to respectively 163 ml min^−1^ m^−2^ (SD 83 ml min^−1^ m^−2^) at T2 (*p* < 0.01) and 142 ml min^−1^ m^−2^ (SD 70 ml min^−1^ m^−2^) at T3 (*p* < 0.01). The effect of NE on PVFi is less clear and no significant alternations are seen.

**Table 4:** Hepatic blood flow & pressure measurements

Variable	Timepoint	Total Group (*n* = 28)	Group S (*n* = 20)	Group NS (*n* = 8)
Total HBF indexed (ml min^−1^ m^−2^)	T1	548 (182)	511 (168)	640 (191)
	T2	465 (149)^#^	447 (153) ^#^	508 (138)^#^
	T3	458 (144)^#^	454 (145) ^#^	467 (149)^#^
Relative Total HBF (% of CO)	T1	17.8 (5.7)	16.7 (5.6)	20.4 (5.4)
	T2	15.2 (4.6)^#^	14.7 (4.9) ^#^	16.3 (3.8) ^#^
	T3	14.6 (4.3)^#^	14.6 (4.5) ^#^	14.7 (4.0)^#^
HAF indexed (ml min^−1^ m^−2^)	T1	215 (118)	216 (128)	212 (95)
	T2	163 (83)^#^	162 (82)^#^	164 (92)^#^
	T3	142 (70)^#^	150 (79)^#^	124 (41)^#^
Relative HAF (% of CO)	T1	6.9 (3.6)	7.0 (3.9)	6.8 (3.0)
	T2	5.3 (2.7)^#^	5.3 (2.5)^#^	5.3 (2.9)^#^
	T3	4.6 (2.3)^#^	4.8 (2.6)^#^	3.9 (1.3)^#^
Conductance HAF (ml min^−1^ m^−2^ mmHg^−1^)	T1	3.0 (1.8)	3.0 (1.9)	3.0 (1.4)
	T2	1.9 (1.0)^#^	1.9 (0.9)^#^	2.0 (1.1)^#^
	T2	1.6 (0.8)^#^	1.6 (0.9)^#^	1.3 (0.4)^#^
PVF indexed (ml min^−1^ m^−2^)	T1	333 (137)	295 (109)^*^	429 (161)^*^
	T2	302 (117)	285 (109)	344 (133)^#^
	T3	315 (120)	304 (109)	344 (151)^#^
Relative PVF (% of CO)	T1	10.9 (4.5)	9.8 (4.0)^*^	13.7 (4.8)^*^
	T2	9.9 (3.7)	9.4 (2.5)	11.1 (3.8)^#^
	T3	10.0 (3.5)	9.7 (3.3)	10.8 (4.2)^#^
Portal Pressure (mmHg)	T1	10 (5)	9 (5)	11 (5)
	T2	11 (5)	10 (5)	12 (5)
	T3	10 (5)	9 (5)	12 (6)
Caval Pressure (mmHg)	T1	7 (4)	6 (3)	8 (4)
	T2	6 (4)	6 (4)	6 (4)
	T3	7 (4)	7 (4)	6 (4)
PI Portal Vein	T1	0.6 (0.4)	0.7 (0.5)	0.5 (0.3)
	T2	1.2 (2.3)	1.4 (2.7)	0.7 (0.4)
	T3	0.6 (0.3)	0.6 (0.3)	0.6 (0.3)
PI Hepatic Artery	T1	1.2 (0.5)	1.3 (0.6)	1.0 (0.4)
	T2	1.5 (0.5)	1.6 (0.5)	1.4 (0.5)
	T3	1.5 (0.7)	1.6 (0.6)	1.4 (0.8)

Data are expressed in mean (SD)

*HBF* Hepatic Blood Flow, *HAF* Hepatic Arterial blood Flow, *PVF* Portal Vein blood Flow, *PI* Pulsatility Index

Significant differences are marked as * for significant between group difference and ^#^ for significant within group difference, compared to T1 (*p* < 0.05)

### Hepatic vascular pressure and resistance

Caval and portal pressures remained unchanged with NE infusion. NE significantly decreased conductance from 3.0 ml min^−1^ m^−2^ mmHg^−1^ (SD 1.8 ml min^−1^ m^−2^ mmHg^−1^) at T1 to respectively 1.9 min^−1^ m^−2^ mmHg^−1^ (SD 1.0 ml min^−1^ m^−2^ mmHg^−1^) at T2 (*p* < 0.01) and 1.6 min^−1^ m^−2^ mmHg^−1^ (SD 0.8 ml min^−1^ m^−2^ mmHg^−1^) at T3 (*p* < 0.01), indicating its effect on the vascular tone of the hepatic artery. The PVRi, however, remained similar over time. Additionally, the PI remained identical.

### Interaction with somatostatin

**Fig. 2:**
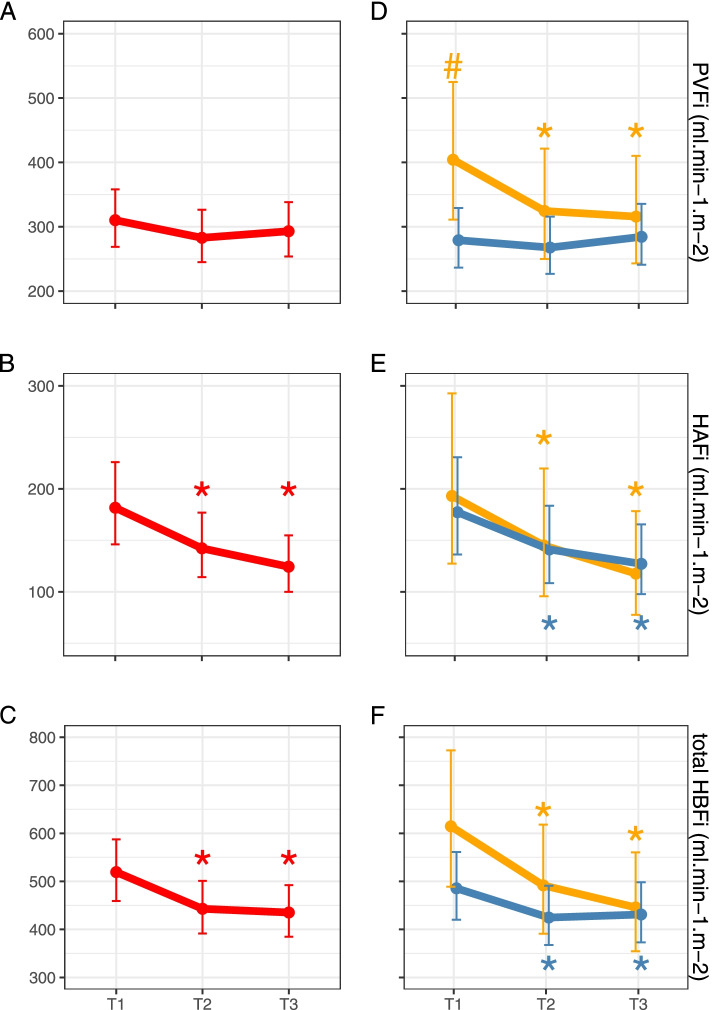
Modelled impact of norepinephrine on hepatic blood flows. Left: graphical representation of the model for PVFi (**A**), HAFi (**B**) and total HBFi (**C**). The red lines represent the results for the total patient group. Right: updated model incorporating interaction for somatostatin for PVFi (D), HAFi (**E**) and total HBFi (**F**). The blue lines represent the results for the somatostatin-treated patients (group S), compared with the orange lines which represent the results of the untreated patients (group NS). Portal vein flow indexed (PVFi), hepatic arterial flow indexed (HAFi) and total hepatic blood flow indexed (HBFi). # Statistically significant difference between groups at T1, * statistically significant difference compared with T1 within groups

Post-hoc analysis showed that SOMATO interacts with this underlying mechanism. NE dose-dependently reduced HAF_i_ in both groups. For PVF_i_ however, effects of NE differed between group S and group NS. Patients receiving SOMATO (group S) had a significantly lower PVF_i_ of 295 ml min^−1^ m^−2^ (SD 109 ml min^−1^ m^−2^) versus 429 ml min^−1^ m^−2^ (SD 161 ml min^−1^ m^−2^) at T1 (*p* < 0.05), which remained unchanged with NE. In untreated patients (group NS), NE decreased PVF_i_ and the reduction of total HBFi is the result of a reduction in HAFi and PVFi. Data are summarized in Table 4 and Fig. 2.

## Discussion

The results of the current study show that in patients under a strict GDHT protocol, administration of NE resulted in significantly reduced total HBF which was mediated by a reduction in HAF while the effect on PVF remained unclear. A post-hoc analysis showed that this response seemed to be influenced by the administration of SOMATO. SOMATO-treated patients had a lower PVF at baseline and administration of NE in these patients, lead to a reduction of total HBF primarily by a reduction of HAF while PVF remained low and unchanged. In SOMATO-untreated patients, total HBF reduction was mediated by both a reduction in HAF and PVF.

We choose pancreaticoduodenectomy as experimental model for this study because – in contrast to liver surgery – this type of surgery is standardized without potential anticipated hemodynamic disturbances. Additionally, there is an easy access to hepatic vasculature during the procedure, which allowed us to perform direct flow measurements of HBF. No previous human studies have used direct flow measurements in assessing the effect of NE on HBF. TTFM was used, which is very reliable and considered to be the ¨gold standard¨ for measuring blood flow [16].

Recent studies emphasize the importance of maintaining adequate blood pressure to prevent postoperative organ failure [1, 2]. Heart, brain and kidney have a robust autoregulation, in which the intrinsic regulatory mechanism provides adequate blood flow despite fluctuations in blood pressure [21], while in the liver, this autoregulatory protection mechanism is weak [14].

NE is frequently used as vasopressor to counteract hypotension during high-risk surgery or critically ill patients [3, 22]. It is used to increase blood pressure, but less attention is given to the potential important effects of NE on regional blood flow.

NE could play a dual role, on the one hand by modulating macro-hemodynamic variables such as blood pressure and cardiac function and on the other hand by affecting regional HBF. Previous animal studies have shown that NE improves cardiac output and venous return by recruitment of unstressed volume into stressed volume [7, 23, 24]. Small human studies in septic patients have confirmed this [25]. The volume status of the patient seems to be of particular importance for this effect, as it was primarily observed in hypervolemic patients who received higher dosage of NE [24, 26]. In our study, such finding was not observed, and CI remained unchanged. This may be the consequence of the fact that our patients were already hemodynamically stabilized with low filling pressures and a balanced volume status. Consequently, to increase the MAP to our target, only small dosages of NE were necessary to obtain the desired effect. Therefore, dosages of NE may have been too low to observe substantial systemic hemodynamic effects. The optimal target for intra-operative MAP is still under debate [1, 2]. The target MAP used in this study, was in the range of the preoperative MAP value. A previous study suggested that target blood pressure is probably best based on the preoperative blood pressure of each individual patient [3].

The effect of NE on splanchnic circulation is puzzling and not yet fully understood. NE affects both systemic and regional hemodynamic variables but the clinical effect of NE on splanchnic vasculature depends on different factors. Relative density of α_1_-, α_2_- and β_2_-adrenorecptor in the splanchnic vasculature, the dosage of NE, the pre-existing sympathetic tonus of the splanchnic vasculature and the blood volume in the splanchnic circulation all may play a crucial role to predict the effect of NE [13].

NE affects the splanchnic vascular resistance sites, which are scattered all over the pre-portal arterial, hepatic arterial and portal venous vasculature [12, 13]. Changes in vascular tone, by increasing resistance, results in a decreased conductance and decreased blood flow [14, 18]. Previous animal studies have shown that NE infusion results in a reduction of total HBF [27], by reducing both HAF and PVF [27, 28]. A small human study confirmed these results [29]. However, another experimental study failed to observe any effect on HBF when NE was used to correct hypotension [26]. Our in vivo study confirmed the results of previous animal studies [27, 28]. Indeed, NE resulted in a reduction of total HBF and due to the weak autoregulatory mechanism, blood flow was not maintained during fluctuations in blood pressure [14, 21]. Thus, the effect of NE on HBF seems to be complex and remains ill-defined.

The effects of NE on hepatic circulation seem to be influenced by the concomitant administration of SOMATO. A total of 20 patients received—on surgical indication – SOMATO, to reduce pancreatic secretions, and protect pancreaticoduodenal anastomosis. Besides reducing pancreatic secretions, SOMATO also induces a mesenterial vasoconstriction [30]. PVF is determined by the outflow of blood from the mesenteric organs and as a result, SOMATO treated patients were shown to have a lower PVF [31, 32]. A post hoc analysis on the results of the current study, confirmed this finding, as patients receiving SOMATO, had lower PVF at baseline, compared with those patients, who had not been treated with SOMATO.

Interestingly, although PVF is lower at baseline, these patients did not have higher HAF as would be expected with the hepatic artery buffer response. This effect can buffer up to 70% of the reduced PVF but ultimately this buffer response seems to become exhausted and HAF returns to normal values [9, 33–35].

As SOMATO was given before pancreatectomy at the start of the operation, a possible explanation would indeed be that the buffer response to SOMATO-induced decrease in PVF had already faded away by the time of the experimental protocol, ultimately resulting in the normalization of HAF. Of note, in the SOMATO-treated patients, NE administration did not further reduce PVF, contrary to what was observed in patients not receiving SOMATO. In these patients, the reduction of total HBF was primarily related to a reduction in HAF. The precise underlying reasons for this different response are not clear and the present study does not allow to further elucidate this issue.

Our study has some limitations. First, our measurements were only performed on the HBF, which is only a part of the splanchnic circulation. As PVF is the sum of outflow of blood from the mesenteric organs, alternations in PVF are indirectly caused by the effect of NE on pre-portal arterial and portal vein vasculature.

Secondly, our measurements were only performed for a short duration of NE infusion, and it remains unclear, how prolonged infusion of NE may alter HBF over time. Further research is therefore needed to evaluate, whether prolonged NE infusion affects hepatic function and clinical outcomes.

Thirdly, our study cohort consisted of a small group of patients. The sample size was determined for the primary objective, HBF. Although NE significantly reduced HBF, the effect on vascular resistance is less clear. According to Ohm`s law, when flow decreases with unchanged portal and caval pressures, the resistance in the portal vein should be increased. However, we did not find this effect. A possible explanation could be that the measurement techniques such as PI and PVRi, have a low sensitivity [36]. The combination of small sample size and low sensitivity of the measurement technique may explain the absence of a clinical effect on these variables.

The results of the current study show that modulating blood pressure with vasopressors such as NE may substantially alter HBF. These findings underscore the importance of considering also potential effects on regional tissue blood flow. Although NE improves blood pressure, it may profoundly affect regional blood flow.

## Conclusion

Administration of NE reduced total HBF, by decreasing HAF, while the effect on PVF remained unclear. SOMATO-treated patients had a lower PVF at baseline, which remained unaffected during NE infusion. In these patients the decrease in total HBF with NE was entirely related to the decrease in HAF. In SOMATO-untreated patients PVF also significantly decreased with NE.

## Data Availability

The datasets used and / or analyzed during the current study are available due to limitations of ethical approval from the corresponding author on reasonable request.

## Abbreviations

ASA: American society of anesthesiologist
BSA: Body surface area
CI: Cardiac index
CVP: Central venous pressure
Eh: Heart performance
GDHT: Goal-directed hemodynamic therapy
HAF: Hepatic arterial flow
HAF_i_: Hepatic arterial flow indexed to body surface area
HBF: Hepatic blood flow
HBF_i_: Hepatic blood flow indexed to body surface area
INR: International normalized ratio
MAP: Mean arterial pressure
P_Cava_: Caval pressure
P_Porta_: Portal pressure
PVF: Portal vein flow
PVF_i_: Portal vein flow indexed to body surface area
Pms: Mean circulatory filling pressure
PVRi: Portal venous resistance indexed to body surface area
SOMATO: Somatostatin
SVRi: Systemic vascular resistance indexed to body surface area
TCI: Target-controlled infusion
TTFM: Transit-time flow measurement
